# No Impact of Enteral Nutrition on Fecal Short-Chain Fatty Acids in Children with Cerebral Palsy

**DOI:** 10.3390/biomedicines12040897

**Published:** 2024-04-18

**Authors:** Dorota Mickiewicz-Góra, Katarzyna Sznurkowska, Arleta Drozd, Anna Borkowska, Maciej Zagierski, Joanna Troch, Karolina Skonieczna-Żydecka, Agnieszka Szlagatys-Sidorkiewicz

**Affiliations:** 1 Department of Paediatrics, Gastroenterology, Allergology & Paediatric Nutrition, Medical University of Gdansk, 80-803 Gdansk, Poland; katarzyna.sznurkowska@gumed.edu.pl (K.S.); anna.borkowska@gumed.edu.pl (A.B.); maciej.zagierski@gumed.edu.pl (M.Z.); joanna.troch@o2.pl (J.T.); agnieszka.szlagatys-sidorkiewicz@gumed.edu.pl (A.S.-S.); 2 Department of Human Nutrition and Metabolomics, Pomeranian Medical University in Szczecin, 70-204 Szczecin, Poland; 3 Department of Biochemical Science, Pomeranian Medical University in Szczecin, 70-204 Szczecin, Poland; karolina.skonieczna.zydecka@pum.edu.pl

**Keywords:** cerebral palsy, percutaneous endoscopic gastrostomy, microbiome, gut, short-chain fatty acids, SCFA, acetate, butyrate, propionate, valerate

## Abstract

Bacteria can impact the host organism through their metabolites, with short-chain fatty acids (SCFAs) being the most important, including acetate (C2), propionate (C3), butyrate (C4), valerate (C5n), and isovalerate (C5i). This study aimed to identify the impact of enteral nutrition on SCFAs in children with cerebral palsy and to test the hypothesis that the type of nutrition in cerebral palsy affects gut SCFA levels. Cerebral palsy is a heterogeneous syndrome resulting from non-progressive damage to the central nervous system. The study group included 30 children diagnosed with cerebral palsy, receiving enteral nutrition (Cerebral Palsy Enteral Nutrition (CPEN)) via gastrostomy. The first reference group (Cerebral Palsy Controls (CPCs)) consisted of 24 children diagnosed with cerebral palsy and fed orally on a regular diet. The second reference group (Healthy Controls (HCs)) consisted of 24 healthy children with no chronic disease and fed on a regular diet. Isolation and measurement of SCFAs were conducted using gas chromatography. Differences were observed in the median contents of isobutyric acid, valeric acid, and isovaleric acid between the CPC group, which had significantly higher levels of those acids than the HC group. No differences were found between the CPEN and CPC groups nor between the CPEN and HC groups. We conclude that enteral nutrition in cerebral palsy has no influence on the levels of SCFAs.

## 1. Introduction

The gut microbiota, also referred to as the microbiome, comprises microorganisms including bacteria, fungi, yeasts, eukaryotic viruses, bacteriophages, and archaea, found mainly in the human intestine, as well as the stomach or mouth [1]. The human intestine is inhabited by about 300 to 500 species of bacteria [2]. About 95% of the human body microbiota resides in the gut, mainly in the colon [3]. The gut microbiota affects many physiological processes and systems within the host human organism, including digestion, the immune system, the nervous system, hormonal homeostasis, neurotransmission, and the nourishment of the intestinal epithelium [4]. There is evidence suggesting associations between dysbiosis and obesity, osteoporosis, colorectal cancer, and inflammatory bowel disease [5,6]. The presence of microorganisms in the human intestine is closely linked to both beneficial and adverse health effects, depending on the species [7]. Microbiota vary among individuals depending on factors such as diet, medication, environment, and lifestyle [1,5]. Numerous studies have investigated the relationship between the microbiota and the type of diet both in the early life stages (breastfeeding/formula feeding) and throughout the human lifespan (such as a vegetarian diet versus high-processed foods) [7,8]. Bacteria can impact the host organism through their metabolites of which the most important are short-chain fatty acids (SCFAs). The presence of bacteria is essential in the production of SCFAs. Short-chain fatty acids can be formed through bacterial fermentation of non-digestible prebiotics and other sources of complex carbohydrates (fiber), oligosaccharides, and glycans [4]. SFCAs have both a regulatory function and an immunological one (including maintenance of the colonic pH and regulation of appetite and metabolism). They strengthen the gut barrier function and also act as a source of energy [9]. Moreover, SCFAs can cross the blood–brain barrier and function as transmitters in the gut–brain axis [8,9]. Currently, the gut–brain axis is under thorough investigation, and its role in cerebral palsy needs to be clarified.

Most studies show that the following three SCFAs are among the most commonly found: acetate (C2), propionate (C3), and butyrate (C4). They are produced in a ratio of 3:1:1, and 90–99% of them are used in the gut [8,10,11,12]. Smaller amounts of valerate (C5n) and isovalerate (C5i) have also been found [13].

Acetate is a satiety modulator (leptin synthesis). It plays a role in cholesterol metabolism and lipogenesis, can be the energy source for the astrocytes (glial cells), and has anti-inflammatory effects [8,11,14]. Propionate can be utilized by the liver as a substrate for gluconeogenesis, and it also reduces inflammation. Butyrate has a role in the synthesis of ATP as well as being the source of energy for colonocytes. It can also control gene expression, as well as reduce pro-inflammatory cytokines and support mucosal integrity [8,11,12,14]. Valerate and isovalerate can suppress autoimmunity and lower arterial blood pressure, but their effect on the gut has not been fully investigated [14]. Each of the SCFAs can be produced by different species of bacteria [7,9,13], which are shown in Table 1.

Cerebral palsy (CP) is a heterogeneous syndrome resulting from non-progressive damage to the central nervous system in the fetus or infant, as well as in early childhood up to the age of 2 [15]. It is one of the most common causes of disability in children [16]. Numerous factors causing brain damage lead to abnormalities in the child’s motor development, posture, and motor functions that affect proprioception and can also disturb the development of cognitive functions [17,18]. Due to gastrointestinal motility disorders, such as dysphagia, cerebral palsy often leads to malnutrition [19]. One of the ways to ensure a proper nutritional state in children with CP is long-term enteral feeding with the use of gastrostomy, which can help maintain appropriate energy intake and nutrient levels [19]. The influence of cerebral palsy on gut microbiota and particularly on SCFAs is poorly investigated, but it can be speculated that GI motility disorders, lack of normal motor activity, lack of a varied diet, and undernutrition could all have an impact on the gut microbiota and metabolome.

We aimed to compare a group of children fed enterally with foods for special medical purposes (FSMPs) with the control groups fed a regular diet. Identifying the difference between children with CP and healthy children was another objective of the study.

## 2. Materials and Methods

### 2.1. Participants

The study group included 30 children (F = 17, M = 13) diagnosed with cerebral palsy, receiving enteral nutrition (Cerebral Palsy Enteral Nutrition (CPEN)) via gastrostomy, who were patients of the Outpatient Nutrition Clinic of the Copernicus Hospital in Gdansk and the Department of Paediatrics, Gastroenterology, Allergology & Paediatric Nutrition at the Medical University of Gdansk. There were two reference groups. The first reference group (Cerebral Palsy Controls (CPCs)) consisted of 24 children (F = 9, M = 15) diagnosed with cerebral palsy and fed orally on a regular diet, while 24 healthy children (F = 11, M = 13) with no chronic disease and fed on a regular diet were enrolled in the second reference group (Healthy Controls (HCs)).

The study group inclusion criteria included written caregiver consent, age below 18 years old, diagnosis of cerebral palsy, enteral nutrition with commercial diets, no antibiotic therapy in the period of 3 months preceding the date of the stool sample collection, and no probiotics or prebiotics intake in the period of 3 months preceding the date of the stool sample collection. The reference group inclusion criteria included written caregiver consent, age below 18 years old, diagnosed cerebral palsy, no diagnosed chronic diseases of the digestive system, oral nutrition with a regular diet, no antibiotic therapy in the period of 3 months preceding the date of the stool sample collection, and no probiotics or prebiotics intake in the period of 3 months preceding the date of the stool sample collection. The inclusion criteria are summarized in Table 2 below.

Group demographic characteristics are presented in Table 3.

In the CPEN group, 100% of the children were exclusively fed on a commercial diet, while 83.33% of children were fed on a normocaloric diet and 16.67% on a hypercaloric diet. A total of 26.6% of the CPEN group received a high-fiber diet. In the CPC group, 100% of the participants were fed on a regular diet, 95.83% were fed on a normocaloric diet, and 4.17% on a hypercaloric diet. In the HC group, 100% of the participants received a regular diet, while 79.17% received a normocaloric diet and 20.83%, a hypercaloric diet.

### 2.2. Procedures

After noting the children’s medical history, basic anthropometric measurements (body weight and length/height) were performed. Body weight was measured in the morning, on an empty stomach, using the weight function of the inBody120 body composition analyzer (InBody Co., Ltd. South Korea Manufacturers, Seoul, Republic of Korea). The measurement was made with an accuracy of 100 g. Length measurement was carried out using a stadiometer for standing children and an anthropometric measuring tape for children with a limited ability to maintain a standing position. The result was recorded with an accuracy of 1 mm. Difficulties during the measurement were encountered in children with spasticity of the limbs, for whom the length measurement had to be completed lying down, taking into account the curvature of the body. In these cases, according to recommendations, 7 mm was subtracted from the result [20]. Each measurement was performed three times to obtain a calculation of the average. On this basis, the BMI was calculated in order to establish BMI percentiles, and the results were checked according to the Development Standards for Children and Adolescents aged 6–18 [21], and in children under 5, using the WHO Growth Standards. The distribution of standard deviations (SDs, z-score) was used to assess nutritional status [22]. Gross motor skills were assessed based on the international GMFCS (Gross Motor Function Classification Scale) presented in Table 4 below [23]. The GMFCS clearly describes the gross motor skills of a child affected by CP at different ages. It is not applicable to healthy children.

### 2.3. Fecal Sample Collection

Prior to collecting the stool sample, the parents were trained in how to properly collect it. The stool samples were collected by patients’ caregivers in a clean container to prevent contamination of the sample. After the stool sample was given to the examiner, it was immediately frozen at −80 °C.

### 2.4. Isolation and Measurement of SCFAs Using Gas Chromatography

For each measurement, the stool sample (0.5 g) was unfrozen and suspended in 2.550 μL of distilled water. Then, 300 μL of internal standard (formic acid with an initial concentration of 10 mM) and 150 μL of 2 M HCl solution were added, thus bringing the sample to a pH range of 2–3. The final sample volume was 3 mL. The sample prepared in this way was thoroughly mixed, then shaken for 10 min and centrifuged for 20 min at 5000 rpm. The supernatant was then filtered through a 25 mm/0.45 μm PES syringe filter directly into a chromatography vial.

In order to measure the SCFAs, chromatographic analysis was performed using an Agilent Technologies 1260 System gas chromatograph with a flame ionization detector (FID). The separation of the compounds analyzed was performed using a DB-FFAP column (30 μm 0.53 mm 0.5 μm) (Perlan, cat. no. 125–3237). The injection volume was 1 μL. The carrier gas was hydrogen. The flow rate was 14.4 mL/min.

The thermal profile of the reaction was as follows: the initial temperature (100 °C) was maintained for 0.5 min, then increased to 180 °C at a rate of 8 °C/min and maintained for 1 min. The temperature was then increased to 200 °C (at a rate of 20 °C/min) and maintained for the next 5 min. The total analysis time for one sample was 17.5 min. The fatty acids were identified by comparing their retention times with commercially available standards using ChemStation Software (Agilent Technologies, Revision B.04.02, May 2010, P/N G2070-60135, Cheadle, UK).

The following SCFAs were analyzed: acetic acid (C2), propionic acid (C3), isobutyric acid (C4i), butyric acid (C4n), isovaleric acid (C5i), valeric acid (C5n), isocaproic acid (C6i), and caproic acid (C6n).

The fraction contents of individual short-chain fatty acids were expressed in micromolar percentages (micromole%). Thus, the total of all SCFAs identified added up to 100% in each subject.

The content of C2, C3, and C4 SCFAs (measured separately) and the total content of C2, C3, and C4 (summed up) were calculated and expressed in micromole%. We decided to analyze butyric acid (C4n) and isobutyric acid (C4i) as one acid (both straight and branched forms). The ratio of 3:1:1 for C2:C3:C4 was regarded as normal and 95% of the total SCFA content was considered as optimal as per recommendations [9]. The percentage of patients who met the criteria was calculated for each group.

### 2.5. Statistical Analysis

The normality of the distribution of continuous variables was verified using the Shapiro–Wilk test. Due to significant deviations from a normal distribution, median and interquartile ranges were used to describe continuous variables. Qualitative variables were reported using counts and percentages. Accordingly, statistical testing was based on a non-parametric test, i.e., the Kruskal–Wallis test. Post hoc analyses were performed using the Conover method. Spearman rank correlations were used for continuous variables. A two-sided *p* = 0.05 was taken as the level of significance. The analyses were performed in MedCalc version 22.013 (Ostend, Belgium). To calculate the molar ratios of the SCFAs, Microsoft Excel 2023 PL was used (basic parameters such as median, mean, and standard deviation were calculated). To establish the number of patients who met the recommended criteria, the “countif” and “countifs” functions were used.

## 3. Results

### 3.1. Levels of SCFAs in Fecal Samples

**Figure 1:**
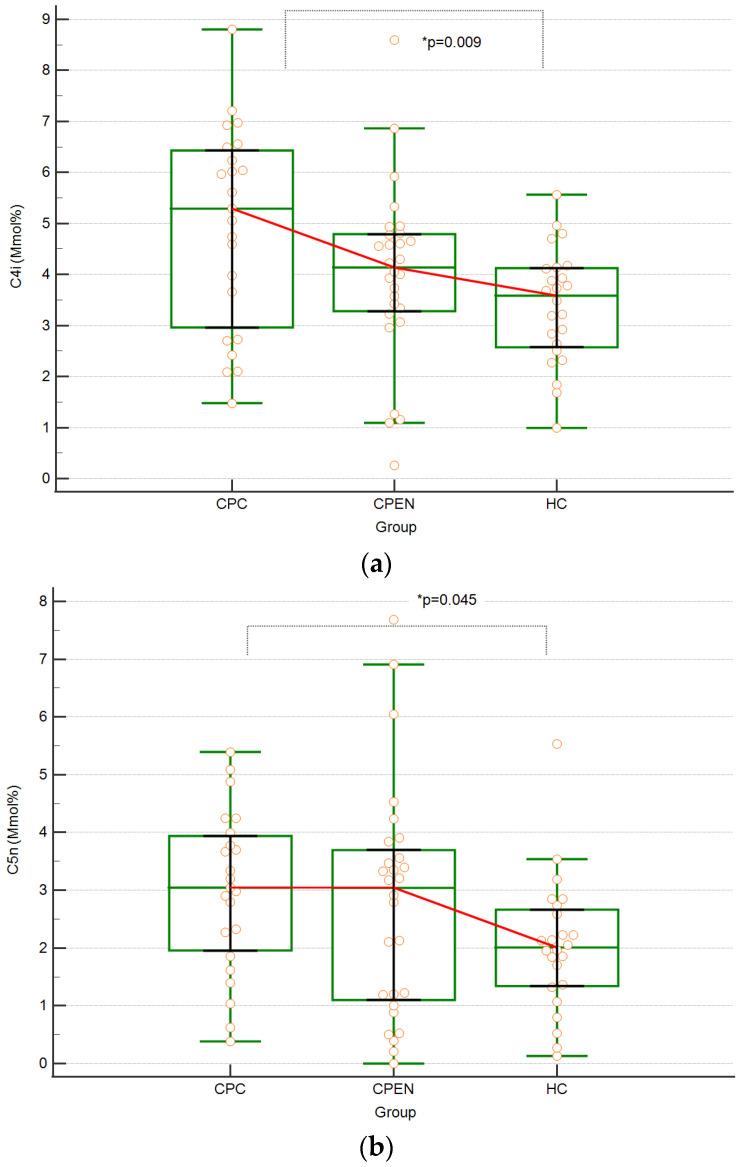

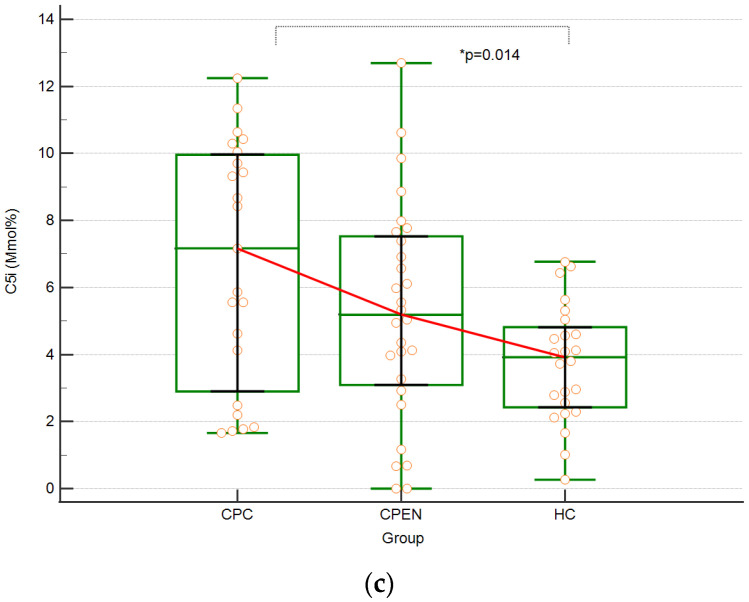
(**a**) Distribution of isobutyric acid (C4) levels in the study and control groups, with medians (indicated by red connecting lines), interquartile ranges, and individual cases highlighted by orange circles. (**b**) Distribution of valeric acid (C5n) levels in the study and control groups, with medians (indicated by red connecting lines), interquartile ranges, and individual cases highlighted by orange circles. (**c**) Distribution of isovaleric acid (C5i) levels in the study and control groups, with medians (indicated by red connecting lines), interquartile ranges, and individual cases highlighted by orange circles; * *p* (*p*-value)—statistically significant difference.

The median level of isobutyric acid (C4i) was significantly higher in CPCs than in HCs (*p* = 0.01). Detailed numerical data including median, range, mean, and SD are shown in Table 5. No differences were found between the CPEN and CPC groups and the CPEN and HC groups. The differences between groups in each pair are shown in Figure 1a.

The median level of valeric acid (C5n) was significantly higher in CPCs than in HCs (*p* = 0.04) (Table 5). No differences were found between the CPEN and CPC groups and the CPEN and HC groups. The differences between groups in each pair are shown in Figure 1b.

The median level of isovaleric acid (C5i) was significantly higher in CPCs than in HCs (*p* = 0.01) (Table 5). No differences were found between the CPEN and CPC groups and the CPEN and HC groups. The differences between groups in each pair are shown in Figure 1c.

No statistically significant differences were found between the groups in the levels of acetic acid (C2, *p* = 0.08), propionic acid (C3, *p* = 0.15), or butyric acid (C4n, *p* = 0.1). The raw SCFAs are shown in Table 5.

No statistically significant relationships were found between SCFA levels and age, BMI, or GMFCS within the groups.

### 3.2. Molar Ratios between SCFA

The proportion of each SCFA and the combined levels of C2, C3, and C4 in the study and control groups are presented in Table 6. The numbers and percentages of patients with abnormal results that did not meet the recommended criteria are shown in bold in Table 6.

The CPEN group contained the lowest proportion of patients with an abnormal (lower than recommended) total percentage of C2 + C3 + C4. The lowest percentages of patients with abnormal results for C2, C3, and C4 were found in the HC, CPEN, and CPC groups, respectively.

In the CPEN, CPC, and HC groups, no patients met all the recommended criteria (95% total content of C2 + C3 + C4 in the 3:1:1 ratio).

## 4. Discussion

The microbiome and its metabolites are currently under extensive investigation. Researchers are finding new effects of the gut microbiota and metabolome on various organism functions. SCFAs are one of the best-studied bacteria metabolites. Alterations in the concentration and composition of SCFAs have been found in many diseases. To the best of our knowledge, our study is the first to assess fecal SCFA levels in children with cerebral palsy. Additionally, publications addressing this issue in other pediatric patients are rather sparse, and their results are inconclusive. Some of these studies concern inflammatory bowel disease (IBD), the condition in which dysbiosis is postulated to contribute to the pathogenesis [24,25].

Treem et al. [25] demonstrated significantly lower concentrations of acetate and higher concentrations of butyrate but no significant difference in total fecal SCFA in children with IBD compared to those in healthy controls. Moreover, these differences correlated with disease activity, although it is not clear if they were a cause or an effect of the inflammation. Some differences were also noted between the two forms of IBD: ulcerative colitis (UC) and Crohn’s disease (CD). Patients with CD had increased concentrations of isobutyrate and isovalerate compared to children with UC [25]. These results contradict Kaczmarczyk, who found a lower concentration of three SCFAs (acetic acid, butyric acid, and valeric acid) in patients with CD compared to healthy controls [26]. Another study concerning SCFAs in pediatric IBD patients, which also assessed the fecal microbiome, was completely inconclusive because of an extremely small study sample [27]. Changes in gut microbiota and short-chain fatty acids (SCFAs) have also been reported in adults with inflammatory bowel disease (IBD) but with conflicting results [28]. Some very interesting data come from studies concerning the impact of exclusive enteral nutrition (EEN), one of the treatment options for Crohn’s disease, on gut microbiota and metabolome [29,30].

Gerasimidis [29] reported lower total microbial diversity and lower butyrate in the gut of patients treated successfully with EEN. These differences persisted throughout the period of treatment and were reversed after its completion. Thus, paradoxically, improvements in pediatric Crohn’s disease during enteral nutrition were associated with a decline in presumptively protective gut bacterial species and metabolites [29]. Tjellström obtained results partially consistent with the aforementioned study, but instead of an increase in fecal butyric acid, a decrease in acetic acid was observed [30]. Inspired by the results of these studies, we aimed to identify the impact of enteral nutrition on SCFAs in children with cerebral palsy. Contrary to the studies cited, we observed no difference in SCFAs between the study group of CP patients fed enterally and the patients receiving a regular diet. Such differences could be expected due to the lower levels of dietary fiber or even no fiber in enteral diets. Our results could mean that enteral nutrition is also favorable for children with cerebral palsy from the point of view of the gut metabolome. The only differences we observed were between the cerebral palsy and healthy control groups, and these differences concerned isobutyric acid, valeric acid, and isovaleric acid, whose functions have not been clearly identified yet. Surprisingly, we found higher levels of these SCFAs in CPC children. Considering the abnormal GI motor function in CP patients, one might expect the opposite result, although the existing evidence is inconclusive as to whether the abnormal motility of the bowel has an impact on the gut microbiome and metabolome. The data on fecal SCFAs in irritable bowel syndrome, regarding the “model” GI motility disorder, were reviewed by Luo in 2021 [31]. Based on the existing, nota bene inconsistent, results, the authors concluded that there were no significant differences between the SCFA levels in individuals with IBS and healthy controls. The lower levels of certain SCFAs found in healthy controls in our cohort could be explained by the low quality of their regular diet. It is highly probable that the diet of CPC children was more carefully selected according to nutritional recommendations to prevent malnutrition, which is associated with cerebral palsy. It should be emphasized that malnutrition itself may alter the metabolome, as demonstrated by Kamil et al. [32], Gordon [33], and Monira [34]. In our study, however, we did not find any correlation between BMI and SCFA levels.

As mentioned earlier, our study is the only one concerning bacteria metabolites in the stools of children suffering from cerebral palsy. There is only one existing study investigating microbiota in CP children, and it should be noted that large discrepancies found between studies on microbiota and metabolome probably result from numerous existing factors including environmental, ethnic, genetic, and pathophysiologic influences on the human gut microbiota. To avoid methodological bias, the study and control groups should be selected in a way that eliminates the influence of factors other than the ones studied. Additionally, in our investigation, healthy individuals on the so-called regular diet were randomly selected for the healthy control group. We regard this as a methodological limitation, although it seems that similar errors have been made by other investigators. In our study, contrary to what was found in the majority of publications, the SCFA levels were expressed in mmol%, which in our opinion reflects better the proportions among them and is independent of stool consistency. The fact that the stool samples were taken by parents at home, introducing the risk of another method-related error, is a minor limitation of our study.

As a separate aspect of our study, we also investigated the ratios of specific SCFA to determine whether their composition in the stool of participant children met the recommended ratio (C2:C3:C4) of 3:1:1. Kaźmierczak-Siedlecka et al. performed a study on 15 participants [35]. They checked mmol% ratios for the three SCFAs: acetate, propionate, and butyrate. Similarly to our research, they found that only rare individuals met the criterion that 95% of the total SCFAs should consist of acetate, propionate, and butyrate in a ratio of 3:1:1. The calculations concerning the proportion of individuals presenting with lower than recommended content of C2, C3, and C4 demonstrated no advantage in any of the groups investigated, CPEN, CPC, and HC.

However, we need to note that the median values of total C2, C3, and C4 were quite close to the recommended values in each group. We conclude that further investigation is needed here because none of the 78 participants of our study met the recommendations. This suggests a possible need to reformulate these recommendations for children.

### Limitations

This study has some limitations. First of all, the study included 30 participants in the CPEN group, whereas a lower number was included in the control groups (CPC = 24, HC = 24). Furthermore, patients were not sex- and age-matched. Another limitation is the fact that food diaries concerning children from the CPC and HC groups only allowed for the approximate estimation of the total calorie intake and did not allow for the estimation of macronutrients or fiber intake. This was due to the fact that processing information (e.g., peeling of fruit or vegetables) and specific information about products (e.g., the type of cereal) was often missing from the diaries.

## 5. Conclusions

Enteral nutrition in cerebral palsy was found to have no influence on the levels of short-chain fatty acids, suggesting a beneficial role in maintaining physiological balance in the microbiota and metabolome. We do not fully understand the significance of our findings in this context, but it may lead to the conclusion that since healthy children have a similar SCFA profile to children fed on a commercial enteral diet through a gastrostomy, it seems that this is both adequate and beneficial for children with cerebral palsy in terms of maintaining a normative content of fatty acids resembling that obtained from samples taken from healthy children.

The higher levels of butyric, valeric, and isovaleric acid in children with cerebral palsy on a regular diet compared to healthy controls may indicate the impact of the disease on the composition of stool short-chain fatty acids.

These observations need to be verified in a larger cohort.

## Figures and Tables

**Table 1:** Species of bacteria producing individual SCFAs.

SCFA	Producers
Acetate (C2)	Butyrate and propionic acid conversion with the participation of *Acetobacterium*, *Acetogenium*, *Eubacterium*, *Clostridium* spp. [9] *Akkermansia muciniphila*, *Bacteroides* spp., *Bifidobacterium* spp., *Provotella* spp., *Ruminococcus* spp., *Blautia hydrogenotrophica*, *Streptococcus* spp. [6]
Propionate (C3)	*Bacteroidetes*, *Propionobacterium* Acetate conversion with the participation of *Firmicutes* [9] *Bacteroides* spp., *Dialister* spp., *Veilonella* spp., *Salmonella* spp. [6] *Coprococcus comes*, *Coprococcus eutactus*, *Anaerostipes* spp., *Coprococcus catus*, *Eubacterium rectale*, *Eubacterium hallii*, *Fecalibacterium prausnitzi*, *Roseburia* spp.
Butyrate (C4)	*Clostridium leptum. Roseburia* spp., *Fecalibacterium prausnitzi*, *Coproccus* spp., *Fusobacterium*, *Eubacterium* Acetate conversion with the participation of *Firmicutes* [9] *Coprococcus comes*, *Coprococcus eutactus*, *Anaerostipes* spp., *Coprococcus catus*, *Eubacterium rectale*, *Eubacterium hallii*, *Fecalibacterium prausnitzi*, *Roseburia* spp. [6]
Valerate (C5)	*Megasphaera elsdenii* [7]

**Table 2:** Group inclusion criteria.

Group	Presence of Cerebral Palsy	Enteral Feeding
CPEN	+	+
CPC	+	−
HC	−	−

CPEN—Cerebral Palsy Enteral Nutrition; CPC—Cerebral Palsy Control; HC—Healthy Control.

**Table 3:** Group demographic characteristics.

Characteristics	CPEN	CPC	HC
Age	10.80 (±3.96)	8.96 (±4.38)	8.08 (±4.34)
Length/Height (cm)	131.18 (±16.65)	122.96 (±25.30)	127.56 (±29.31)
Body mass (kg)	22.92 (±5.95)	22.69 (±13.66)	32.30 (±21.90)
BMI	13.30 (±2.28)	13.93 (±3.30)	17.50 (±3.90)
GMFCS	4.767 (±0.50)	4.21 (±0.88)	No data

CPEN—Cerebral Palsy Enteral Nutrition; CPC—Cerebral Palsy Control, HC—Healthy Control. GMFCS—Gross Motor Function Classification Scale.

**Table 4:** Gross Motor Function Classification Scale (GMFCS).

Level	Description
Level I	Walks without limitations
Level II	Walks with limitations
Level III	Walks with a hand-held mobility device
Level IV	Requires physical assistance or uses powered mobility
Level V	Transported in a manual wheelchair in all settings

**Table 5:** SCFA levels in the study and control groups.

Group		Acetic Acid (C2) [mmol%]	Propionic Acid (C3) [mmol%]	Isobutyric Acid (C4i) [mmol%]	Butyric Acid (C4n) [mmol%]	Isovaleric Acid (C5i) [mmol%]	Valeric Acid (C5n) [mmol%]
CPEN	Median	57.132	16.989	4.137	13.209	5.186	3.04
	Range	41.15–85.593	1.156–26.126	0.259–8.589	1.737–24.353	0–12.696	0–7.686
	Mean ± SD	58.194 ± 9.775	15.969 ± 5.249	4.006 ± 1.731	13.805 ± 5.225	5.251 ± 3.246	2.775 ± 1.992
CPC	Median	53.617	14.993	5.29	15.346	7.169	3.045
	Range	43.973–64.512	9.951–20.097	1.479–8.804	9.122–31.099	1.758–12.247	0.384–5.395
	Mean ± SD	53.739 ± 6.032	15.008 ± 2.713	4.942 ± 1.977	16.577 ± 4.646	6.745 ± 3.618	2.99 ± 1.392
HC	Median	58.902	16.012	3.585	15.398	3.921	2.01
	Range	44.128–71.315	0.55–24.31	0.992–5.565	6.705–27.192	0.263–6.77	0.131–5.538
	Mean ± SD	57.979 ± 5.781	16.995 ± 3.608	3.39 ± 1.1202	15.852 ± 4.228	3.749 ± 1.735	2.036 ± 1.155
*p*-value		*p* = 0.09	*p* = 0.15	*p* = **0.009**	*p* = 0.11	*p* = **0.014**	*p* = **0.045**

CPEN—Cerebral Palsy Enteral Nutrition; CPC—Cerebral Palsy Control; HC—Healthy Control; *p*-value—statistically significant difference (bolded).

**Table 6:** Percentage of C2, C3, and C4 SCFAs in the study and control groups.

Group	(C2 + C3 + C4) Median Range Mean ± SD	C2 Median Range Mean ± SD	C3 Median Range Mean ± SD	C4 Median Range Mean ± SD
Recommendation	95 (%)	3	1	1
CPEN	92.09 (%)	3.15	0.91	0.97
	82.47–100	2.25–4.27	0.06–1.39	0.37–1.70
	91.97 ± 4.80	3.15 ± 0.42	0.87 ± 0.28	0.98 ± 0.31
**Lower than recommended**	**64.29% (*n* = 18)**	**42.86% (*n* = 12)**	**60.71% (*n* = 17)**	**53.57% (*n* = 15)**
CPC	88.46	2.95	0.85	1.24
	82.87–97.18	2.55–3.35	0.52–1.03	0.79–1.68
	89.93 ± 4.58	2.96 ± 0.21	0.82 ± 0.14	1.22 ± 0.25
**Lower than recommended**	**69.57% (*n* = 16)**	**56.52% (*n* = 13)**	**82.61% (*n* = 19)**	**26.09% (*n* = 6)**
HC	94.44	3.13	0.86	1.02
	89.16–99.21	2.47–0.55	0.55–1.28	0.52–1.42
	94.22 ± 2.47	3.07 ± 0.27	0.90 ± 0.19	1.02 ± 0.24
**Lower than recommended**	**66.67% (*n* = 16)**	**37.5% (*n* = 9)**	**70.83% (*n* = 17)**	**33.33% (*n* = 8)**

CPEN—Cerebral Palsy Enteral Nutrition; CPC—Cerebral Palsy Control; HC—Healthy Control. The percentage and number of patients not meeting the criteria were bolded.

## Data Availability

The data presented in this study are available on request from the corresponding author.
